# Identification of quantitative trait loci and candidate genes for primary metabolite content in strawberry fruit

**DOI:** 10.1038/s41438-018-0077-3

**Published:** 2019-01-01

**Authors:** José G. Vallarino, Delphine M. Pott, Eduardo Cruz-Rus, Luis Miranda, Juan J. Medina-Minguez, Victoriano Valpuesta, Alisdair R. Fernie, José F. Sánchez-Sevilla, Sonia Osorio, Iraida Amaya

**Affiliations:** 1 Department of Molecular Biology and Biochemistry, Instituto de Hortofruticultura Subtropical y Mediterránea “La Mayora”, University of Málaga – Consejo Superior de Investigaciones Científicas (IHSM-UMA-CSIC), Campus de Teatinos, 29071 Málaga, Spain; 2 Max-Planck-Institut für Molekulare Pflanzenphysiologie, Am Mühlenberg 1, 14476 Potsdam-Golm, Germany; 3 Genómica y Biotecnología, Centro de Málaga, Instituto Andaluz de Investigación y Formación Agraria y Pesquera (IFAPA), 29140 Málaga, Spain; 4 Ingeniería y Tecnología Agroalimentaria, Centro Las Torres-Tomejil, Instituto Andaluz de Investigación y Formación Agraria y Pesquera (IFAPA) Alcalá del Río, Sevilla, Spain; 5 Ingeniería y Tecnología Agroalimentaria, Centro de Huelva, Instituto Andaluz de Investigación y Formación Agraria y Pesquera (IFAPA), Huelva, Spain

**Keywords:** Plant sciences, Molecular biology

## Abstract

Improvement of nutritional and organoleptic quality of fruits is a key goal in current strawberry breeding programs. The ratio of sugars to acids is a determinant factor contributing to fruit liking, although different sugars and acids contribute in varying degrees to this complex trait. A segregating F_1_ population of 95 individuals, previously characterized for several fruit quality characters, was used to map during 2 years quantitative trait loci (QTL) for 50 primary metabolites, l-ascorbic acid (L-AA) and other related traits such as soluble solid content (SSC), titratable acidity (TA), and pH. A total of 133 mQTL were detected above the established thresholds for 44 traits. Only 12.9% of QTL were detected in the 2 years, suggesting a large environmental influence on primary metabolite content. An objective of this study was the identification of key metabolites that were associated to the overall variation in SSC and acidity. As it was observed in previous studies, a number of QTL controlling several metabolites and traits were co-located in homoeology group V (HG V). mQTL controlling a large variance in raffinose, sucrose, succinic acid, and L-AA were detected in approximate the same chromosomal regions of different homoeologous linkage groups belonging to HG V. Candidate genes for selected mQTL are proposed based on their co-localization, on the predicted function, and their differential gene expression among contrasting F_1_ progeny lines. RNA-seq analysis from progeny lines contrasting in L-AA content detected 826 differentially expressed genes and identified *Mannose-6-phosphate isomerase, FaM6PI1*, as a candidate gene contributing to natural variation in ascorbic acid in strawberry fruit.

## Selective breeding: The genetic basis of better strawberries

Investigations have yielded early insights into the genetic basis of strawberry taste and nutrition. Such information informs efforts to selectively breed the fruit to maximize these qualities. A team of researchers from Spain and Germany, led by the University of Malaga’s Sonia Osorio and the IFAPA´s Iraida Amaya, found 133 locations within strawberry DNA that correlated to variation in metabolic pathways and desirable traits including acidity, sugar content, and the concentration of l-ascorbic acid (vitamin C). Only a small number of associations persisted over the 2 years of investigations, suggesting that environmental factors also wield a significant influence over the strawberry fruit’s molecular makeup. The team then used their data to identify a series of candidate genes that may be functionally linked to strawberry qualities; however, further research is needed to validate those connections.

## Introduction

Strawberry is one of the most important soft fruit crops in the world and its quality is largely tied to the ripening process. During fruit development, the receptacle experiences auxin-dependent phases of division, expansion and ripening, and consequently sugars, organic acids, and volatiles accumulate. Ripe strawberries are therefore highly valued for their delicate flavor, and also as an important source of sugars, minerals, vitamins and antioxidant compounds^1^. Strawberry (*Fragaria* × *ananassa*) is also an important fruit from an agronomic perspective: its global production in 2016 was over 9.1 million tons (FAOSTAT database; accessed 28 Feb. 2018). Traditionally, both fresh market and the processed food industry have demanded strawberry varieties with improved agronomic characteristics such as increased production, fruit size, firmness, and resistance to stresses. This strict focus has indirectly resulted in an erosion of the genetic and biochemical complexity essential for fruit quality^2^. Over the last decade, consumer preferences have driven breeding efforts to improve fruit flavor, mainly through increasing soluble solids content (SSC) as an approximation of total sugar content and the SSC:titratable acidity (TA) ratio^3^. More recently, breeding has aimed to maintain, or increase, the content of nutraceutical compounds^4^. Indeed, if elevated content of nutritional components is combined with a high flavor standard, consumer fruit consumption could be encouraged^5,6^. Breeding more nutritious and better tasting cultivars can be achieved by introgressing natural trait variation, which requires understanding the heritability of those traits. In this regard, QTL analysis not only provides information on the genetic control of traits useful for breeding new varieties through marker-assisted selection, but also on the relationships among genes influencing the traits.

Metabolite profiling has been successfully used to identify key compounds involved in development, stress tolerance, and nutritional value in many important crops^7,8^. This approach was likewise applied to discover enzyme functions, reconstruct important pathways, and even define their regulation^9,10^. It was also used to explore natural variability in wild species in order to identify valuable germplasm, that could be eventually used for the improvement of agriculturally important crops^11–13^. Additionally, considerable biological insight was obtained from metabolic profiling coupled with genome-wide association studies (GWAS) in important crops including tomato, maize, and rice^14–19^. While its argued that whole-genome regression may be more practical for artificial selection, it is of little help in understanding molecular function. Recent work in tomato strengthened the role of the cell wall invertase *Lin5* as a major determinant of soluble solid content, as well as the gene *E8* as underlying quantitative variance in volatile organic compounds^20^. Quality phenotypic data are necessary requirement for metabolite QTL studies to characterize the gene to metabolite association^21,22^. Elucidation of gene/phenotype associations requires integration of high-quality metabolic data with genomic and genetic studies^21,22^. Metabolic analysis has been increasingly used to assist elite germplasm selection^23,24^. In tomato, the first study using metabolite profiling showed that metabolic traits correlated with phenotypic traits such as yield or harvest index, exposing the challenge to use metabolites as biomarkers^22^. In cultivated strawberry, metabolic traits have been studied using this approach, including the identification of QTL for volatile organic compounds, glucose, fructose, sucrose, malic, citric, and l-ascorbic acids^25–27^. Strawberry fruits are considered a rich source of vitamin C, or l-ascorbic acid (L-AA), a water-soluble vitamin that is an essential dietary component for humans^28^. However, L-AA concentration varies widely between strawberry cultivars and also among wild *Fragaria* species, ranging from less than 10 to more than 80 mg/100 g FW^4,28–30^. Natural variation in L-AA content has been used for the detection of QTL controlling this important trait^26^. In tomato, studies have also identified QTL controlling pigments, cell wall components, sesquiterpenoids, acyl-sugars, and cuticle composition in fruits^31–35^. Furthermore, these studies were helpful in elucidating the biosynthetic pathways of different volatiles such as phenylethanol, phenylacetaldehyde, mesifurane, g-decalactone, and methyl anthranilate^27,32,36–38^, as well as specific glycoalkaloids^39,40^. Such research contributed to enhancement of our understanding of fruit specialized metabolism^41^.

The major aim of this work was to characterize the variation and genetic control of metabolic traits related to primary metabolism in strawberry fruit using an F_1_ mapping population. The population was derived from the cross between two contrasting lines, ‘232’ and ‘1392’ that differed, among other traits, in overall sweetness scores, TA and L-AA content^26^. In a previous report, QTL for agronomical and fruit quality traits were identified in the ‘232’ × ‘1392’ population^26^. Using the same population for analysis of QTL controlling the variation in primary metabolites may allow the identification of common loci affecting mQTL and key fruit quality traits such as SSC, acidity, and volatile organic compounds. To achieve this goal, we firstly applied a well-established gas chromatography coupled to mass spectrometry (GC-MS) platform^42^, examining primary fruit metabolite levels in ripe fruit. Secondly, we evaluated metabolite correlations using clustering methods. Thirdly, we searched for mQTL controlling primary metabolites, and compared them to previously identified QTL for SSC and TA, and finally, we identified candidate genes located in the confidence interval of selected QTL. For the last goal, we took advantage of the high synteny of the genomes of cultivated strawberry (*F.* *×* *ananassa*) and the diploid *Fragaria vesca*, an ancestor of the cultivated species with a recently updated genome sequence available (*Fragaria vesca* v4.0.a1 genome)^43–46^.

## Results

### Variation in the metabolic fruit composition in ‘232’ × ‘1392’ mapping population

This study was based on the evaluation of strawberry fruits harvested from two independent years (2013 and 2014). As an initial approach to assess the variation in primary metabolism in fruits, we investigated which metabolites were present in ripe fruit extracts from parents and F_1_ progeny lines from the cross of selection lines ‘232’ and ‘1392’. This population has been shown to segregate for a wide range of traits, including yield, fruit size, and important fruit quality traits such as SSC, acidity, and volatile organic compounds^26,27^. Metabolites were detected and semi-quantified by gas chromatography-(TOF) mass spectrometry (GC-TOF-MS) using the same protocol as previously described^42^. A total of 50 metabolites were identified, including amino (19) and organic acids (11), soluble sugars (13), sugar alcohols (3), phosphorylated intermediates (2), and other compounds (2) (Supplementary Table 1). The relative content of these 50 primary metabolites in fruits of the parents, and the means and ranges in the F_1_ progeny in the 2 years, are shown in Table 1. A high level of divergence in terms of primary metabolism was evident when comparing metabolite contents between the parental lines. The levels of five of the measured amino acids (valine, β-alanine, tyrosine, aspartic acid, and pyroglutamic acid), two tricarboxylic acid (TCA) cycle intermediates (succinic acid, and 2-oxoglutaric acid), six sugars (raffinose, sucrose, maltose, fucose, rhamnose, 1-kestose), and pyruvic acid were significantly lower in fruits of ‘232’ than in ‘1392’ in the 2 years. By contrast, only the content of xylose was significantly higher in fruits of ‘232’ than in ‘1392’ in both years (Table 1). Interestingly, considerable variation in most of the metabolites was found in the progeny, even for metabolites where no significant differences were found between the parents (Table 1). The amino acids glutamic acid, methionine and tryptophan are examples of metabolites with similar levels in the parental lines but a wide range of variation in the progeny. All identified metabolites displayed continuous variation in the progeny, supporting the quantitative nature of these traits, although they were not normally distributed according to the Shapiro–Wilk test (*p* ≤ 0.05). Only pyruvic acid fitted a normal distribution for both years, while leucine, fucose, and glucose fit a normal distribution only in 2013, and aspartic and glutamic acids and *myo*-inositol only in 2014. In general, primary metabolite distributions were generally skewed toward low values although transgressive segregation was frequently found in both directions (Table 1). Thus, the observed variation in the metabolite levels indicated the suitability of the population for the search of mQTL controlling strawberry fruit composition. However, in order to achieve normality for statistical analyses, relative metabolite content was transformed to logarithm for the majority of them (see statistical methods).

**Table 1:** Relative content of primary metabolites in ‘232’, ‘1392’, and their F_1_ progeny in two consecutive years

	Parental Lines					F1 progeny		
	2013		2014		2013		2014	
Metabolite	232	1392	232	1392	Mean ± SD	Range	Mean ± SD	Range
Alanine	**0.63**	1.00	1.23	1.00	0.91 ± 0.36	0.35–2.33	1.52 ± 0.65	0.29–3.90
Asparagine	0.71	1.00	0.75	1.00	0.86 ± 0.26	0.49–1.92	1.19 ± 0.45	0.48–2.84
Aspartic acid	**0.59**	1.00	**0.69**	1.00	2.66 ± 10.19	0.55–98.27	2.41 ± 3.42	0.40–28.75
Glutamic acid	0.81	1.00	0.80	1.00	1.00 ± 0.35	0.43–2.63	1.76 ± 0.78	0.40–3.60
Glutamine	**0.33**	1.00	0.71	1.00	1.04 ± 0.31	0.45–2.28	1.44 ± 0.37	0.69–3.06
Glycine	**0.55**	1.00	1.05	1.00	1.81 ± 0.68	0.58–4.29	1.50 ± 0.57	0.53–2.77
Isoleucine	1.11	1.00	**0.67**	1.00	0.86 ± 0.63	0.15–2.80	1.22 ± 0.84	0.16–4.33
Leucine	0.92	1.00	**0.72**	1.00	0.78 ± 0.18	0.43–1.25	1.04 ± 0.27	0.54–2.05
Methionine	0.98	1.00	0.85	1.00	1.42 ± 0.69	0.48–4.78	1.17 ± 0.50	0.37–2.98
Phenylalanine	0.88	1.00	**0.49**	1.00	1.03 ± 0.23	0.63–2.27	1.11 ± 0.25	0.71–2.42
Proline	0.95	1.00	**1.69**	1.00	0.89 ± 0.42	0.00–1.98	0.79 ± 0.44	0.00–2.65
Pyroglutamic acid	**0.41**	1.00	**0.39**	1.00	0.78 ± 0.41	0.18–2.15	0.91 ± 0.57	0.23–3.03
Serine	**0.56**	1.00	0.96	1.00	0.80 ± 0.48	0.19–2.34	0.80 ± 0.53	0.14–2.74
Threonine	**0.72**	1.00	0.94	1.00	1.13 ± 0.28	0.66–1.99	1.28 ± 0.38	0.58–2.22
Tryptophan	0.99	1.00	0.80	1.00	0.89 ± 0.41	0.28–2.18	2.27 ± 2.41	0.32–13.72
Tyrosine	**0.40**	1.00	**0.41**	1.00	0.78 ± 0.36	0.11–1.78	**0.91** ± **0.34**	**0.27**–**1.67**
Valine	**0.66**	1.00	**0.72**	1.00	0.85 ± 0.21	0.53–1.71	0.99 ± 0.61	0.00–4.32
β-Alanine	**0.41**	1.00	**0.65**	1.00	0.78 ± 0.31	0.26–2.03	1.27 ± 0.40	0.55–2.30
GABA	**0.69**	1.00	0.86	1.00	0.89 ± 0.31	0.40–2.16	**1.08** ± **0.40**	**0.26**–**2.32**
2-Oxoglutaric acid	**0.12**	1.00	**0.23**	1.00	0.94 ± 0.61	0.31–3.42	1.75 ± 1.00	0.46–5.12
Citric acid	0.82	1.00	1.18	1.00	0.84 ± 0.30	0.33–1.80	1.27 ± 0.68	0.24–4.28
Dehydroascorbic acid	0.76	1.00	0.99	1.00	0.90 ± 0.34	0.32–1.82	1.19 ± 0.60	0.30–3.14
Fumaric acid	**0.52**	1.00	0.84	1.00	0.68 ± 0.28	0.18–1.63	0.83 ± 0.33	0.22–1.86
Glucuronic acid	0.83	1.00	**0.73**	1.00	1.17 ± 0.69	0.29–4.47	1.58 ± 0.77	0.57–5.19
Glyceric acid	1.06	1.00	1.13	1.00	**0.69** ± **0.23**	**0.17**–**1.32**	**0.71** ± **0.25**	**0.28**–**1.13**
Malic acid	**0.66**	1.00	0.85	1.00	1.50 ± 0.63	0.59–3.47	1.11 ± 0.46	0.32–2.42
Phosphoric acid	**0.80**	1.00	1.26	1.00	**0.70** ± **0.12**	**0.48**–**1.02**	0.76 ± 0.18	0.46–1.24
Pyruvic acid	**0.46**	1.00	**0.47**	1.00	0.49 ± 0.21	0.12–1.04	1.13 ± 0.60	0.28–3.15
Quinic acid	0.56	1.00	0.62	1.00	1.23 ± 0.28	0.68–2.58	1.19 ± 0.38	0.61–2.71
Succinic acid	**0.18**	1.00	**0.66**	1.00	0.85 ± 0.53	0.10–3.23	1.11 ± 0.85	0.23–6.00
Threonic acid	0.91	1.00	1.05	1.00	1.11 ± 0.17	0.75–1.70	1.30 ± 0.25	0.82–2.25
1-Kestose	**0.30**	1.00	**0.50**	1.00	0.82 ± 0.45	0.30–2.76	1.37 ± 0.74	0.24.–3.71
Erythritol	0.95	1.00	1.09	1.00	0.76 ± 0.34	0.27–1.94	0.85 ± 0.48	0.24–2.5
Fructose	1.03	1.00	**1.18**	1.00	0.82 ± 0.24	0.32–1.65	**0.88** ± **0.27**	**0.25**–**1.70**
Fructose-6-phosphate	**0.72**	1.00	0.77	1.00	1.29 ± 0.69	0.40–3.85	1.40 ± 0.72	0.45–3.63
Fucose	**0.52**	1.00	**0.54**	1.00	0.64 ± 0.36	0.11–1.87	1.06 ± 0.59	0.23–3.17
Galactinol	**0.62**	1.00	**1.94**	1.00	0.92 ± 1.11	0.00–7.98	1.36 ± 1.14	0.18–5.48
Glucose	1.00	1.00	**1.26**	1.00	0.66 ± 0.23	0.18–1.66	1.50 ± 0.70	0.46–3.38
Glucose-6-phosphate	**0.75**	1.00	1.15	1.00	0.75 ± 0.59	0.00–2.66	1.03 ± 0.68	0.00–3.57
Isomaltose	0.74	1.00	0.98	1.00	1.05 ± 0.38	0.42–2.61	1.17 ± 0.50	0.37–3.38
Maltose	**0.42**	1.00	**0.49**	1.00	0.46 ± 0.37	0.11–1.79	0.85 ± 0.66	0.00–3.27
Maltotriose	**0.54**	1.00	1.04	1.00	0.81 ± 0.35	0.29–2.20	1.04 ± 0.35	0.35–1.83
Myo-inositol	**0.28**	1.00	0.67	1.00	0.68 ± 0.17	0.27–1.30	1.04 ± 0.38	0.51–2.82
Raffinose	**0.35**	1.00	**0.39**	1.00	1.18 ± 0.21	0.68–1.82	1.25 ± 0.41	0.57–2.58
Rhamnose	**0.79**	1.00	**0.70**	1.00	0.81 ± 0.26	0.36–1.84	0.80 ± 0.32	0.19–2.04
Sucrose	**0.54**	1.00	**0.53**	1.00	0.91 ± 0.18	0.51–1.61	1.07 ± 0.26	0.62–2.08
Xylose	**1.34**	1.00	**1.65**	1.00	0.92 ± 0.35	0.56–2.46	0.93 ± 0.39	0.42–3.41
α,α-Trehalose	**0.26**	1.00	0.74	1.00	0.99 ± 0.41	0.41–2.18	0.75 ± 0.25	0.40–1.83
1-*O*-methyl-α-d-glucopyranoside	**0.64**	1.00	0.83	1.00	**0.93** ± **0.10**	**0.61**–**1.13**	1.08 ± 0.33	0.47–3.00
Putrescine	**1.59**	1.00	**0.75**	1.00	1.90 ± 1.95	0.00–9.67	0.95 ± 0.70	0.19–4.48

Metabolite levels which are significantly different between ‘232’ and ‘1392’ lines are indicated in bold in the ‘232’ columns and metabolites with normal distributions are labeled in bold in the corresponding F_1_ columns (*P* < 0.05; Shapiro–Wilks test)

**Fig. 1:**
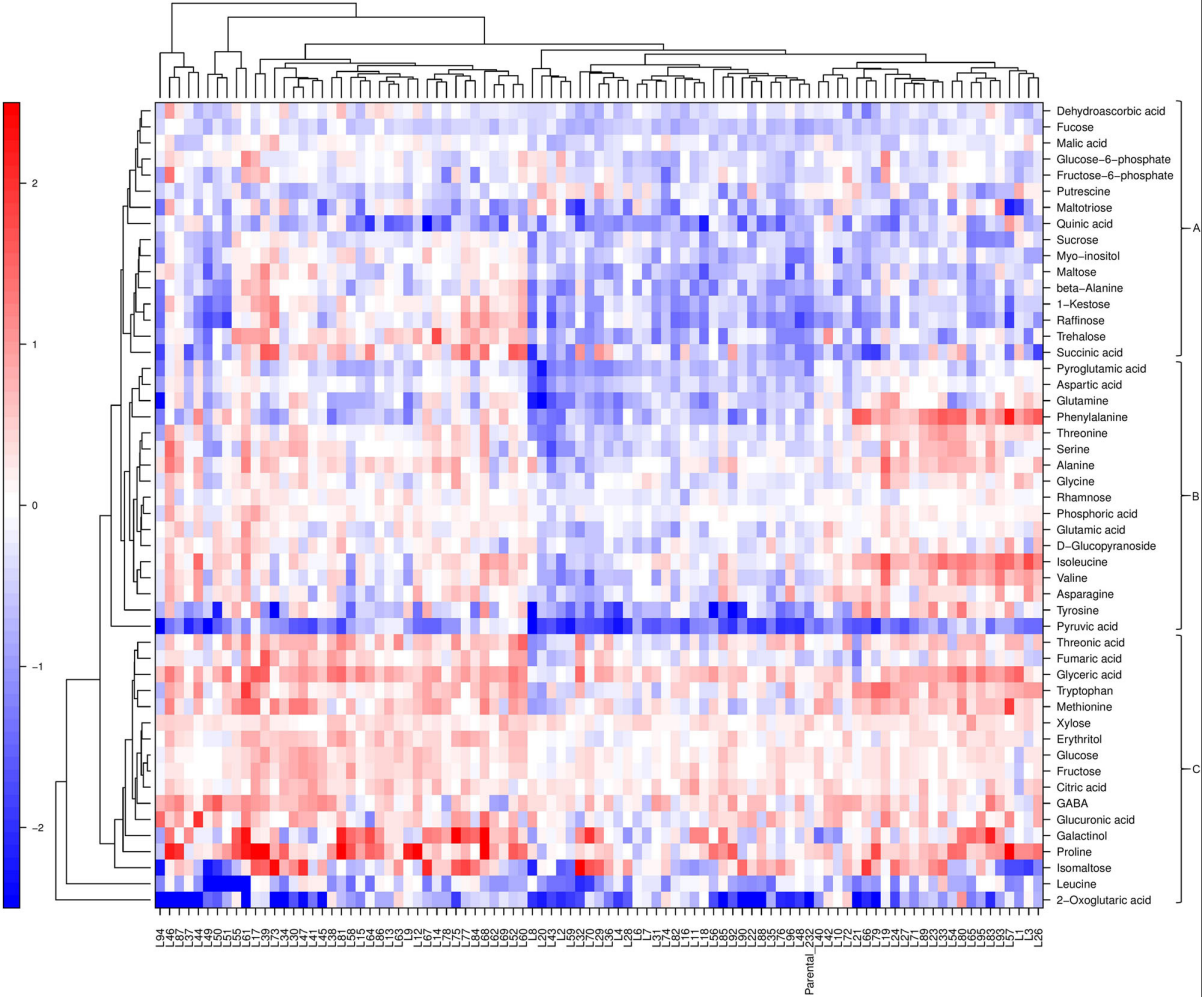
Hierarchical cluster analysis (HCA) and heatmap visualization of averaged metabolite profiles in the ‘232’ × ‘1392’ population over two successive years (2013–2014). F_1_ lines with a relative content for a given compound similar, lower, or higher than that of the reference parent ‘1392’ are shown in white, blue, or red, respectively

Hierarchical cluster analysis (HCA) using relative metabolite content in fruits of parental lines and the F_1_ progeny, harvested in 2013 and 2014, was used to further investigate the relationship between compounds and lines within the population (Fig. 1). The analysis highlighted that the range in variation found for the majority of metabolites among the progeny was much larger than that seen between the two parental lines. Identified metabolites were grouped into three clusters (A–C) of similar size (30–34% of metabolites), each of them containing different classes of metabolites. Interestingly, cluster A was enriched in sugar and sugar-derivatives, namely raffinose, 1-kestose, trehalose, fucose, *myo*-inositol, sucrose, maltose, maltotriose, glucose-6P, and fructose-6P. Cluster B included mainly amino acids such as aspartate, glutamine, phenylalanine, threonine, serine, alanine, glycine, glutamic acid, isoleucine, valine, asparagine, and tyrosine. Cluster C contained more diverse compounds including those from the sugars, amino acids, and organic acids categories (Fig. 1).

### Metabolite correlation analysis

**Fig. 2:**
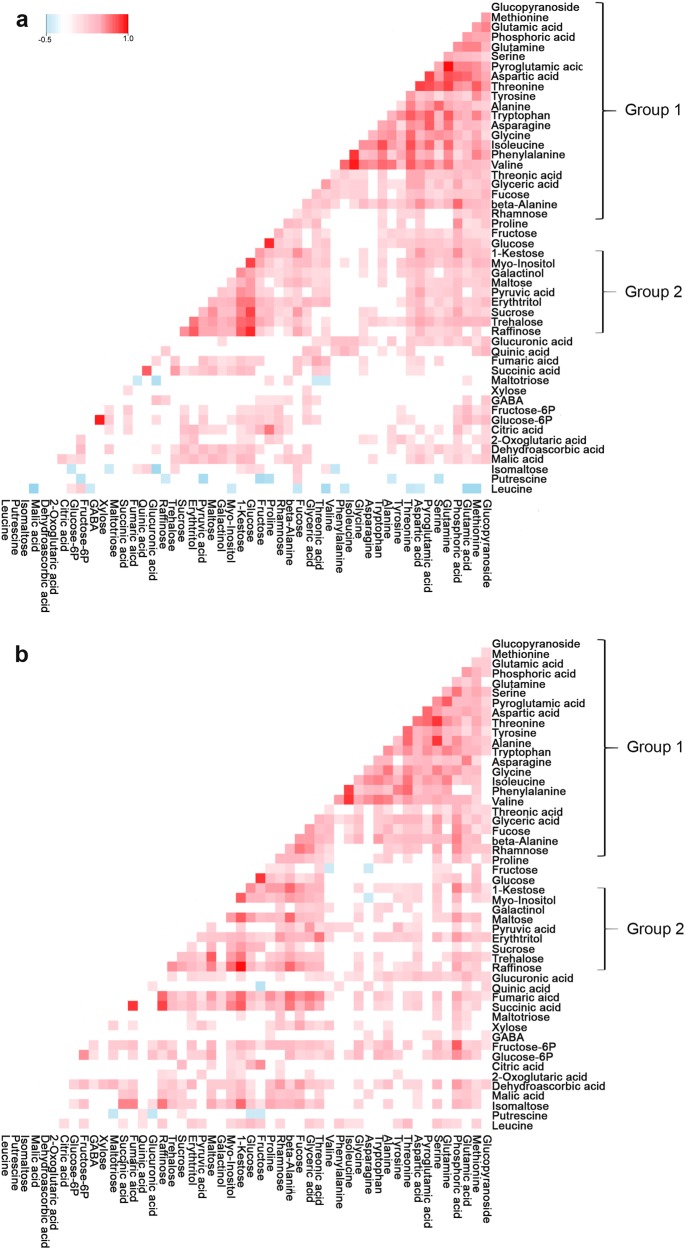
Visualization of metabolite-metabolite correlations. Heat map representation of pair-wise correlations between metabolites identified in ‘232’ × ‘1392’ for years 2013 (**a**) and 2014 (**b**). Each square indicates a given r value resulting from Pearson correlation analysis in a false color scale (red and blue indicate positive and negative correlations, respectively). The self-comparisons are indicated in white

Next, the coordinated metabolic changes were identified by performing a pair-wise correlation analysis using Pearson’s correlation at a permissive stringency threshold (*P* < 0.05; values in Supplementary Table 2). This analysis reflects the degree of coordination of the metabolic changes in the population and facilitates the detection of possible co-regulations among different metabolites. In order to identify strong correlations between the two seasons, the analysis was performed for years 2013 and 2014 separately. A total of 690 significant correlations (*P* < 0.05) were found for the first year (Fig. 2a). Of these, 665 were positive and 50 negative. As expected, the same analysis performed using data from the 2014 year resulted in a similar number of significant correlations (Fig. 2b). Indeed, a total of 705 correlations, of which 697 were positive and only eight were negative in 2014 (Fig. 2b). Interestingly, two groups of metabolites displayed strong correlations in both years. One group (group 1; Fig. 2) included all metabolites grouped in cluster B from Fig. 1 while the second group (group 2; Fig. 2) included sugars and sugar-alcohols from cluster A (Fig. 1). Metabolites in the second group including maltose, sucrose, *myo*-inositol, trehalose, kestose, and raffinose displayed correlations ranging from 0.61 to 0.90 in 2013 and from 0.41 to 0.94 in 2014 (Fig. 2). Fructose and glucose were also included in this group and were highly correlated in both years (0.88 and 0.86, for 2013 and 2014, respectively). Also, strong correlations were found both years for glucose- and fructose-6-phosphate (0.93 and 0.62, respectively), succinic and fumaric acids (0.76 and 0.84, respectively), sucrose and raffinose (0.75 and 0.54, respectively), as well as for raffinose and succinic acid (0.576, 0.819, respectively).

### Identification of mQTL for primary metabolism in strawberry fruits

**Fig. 3:**
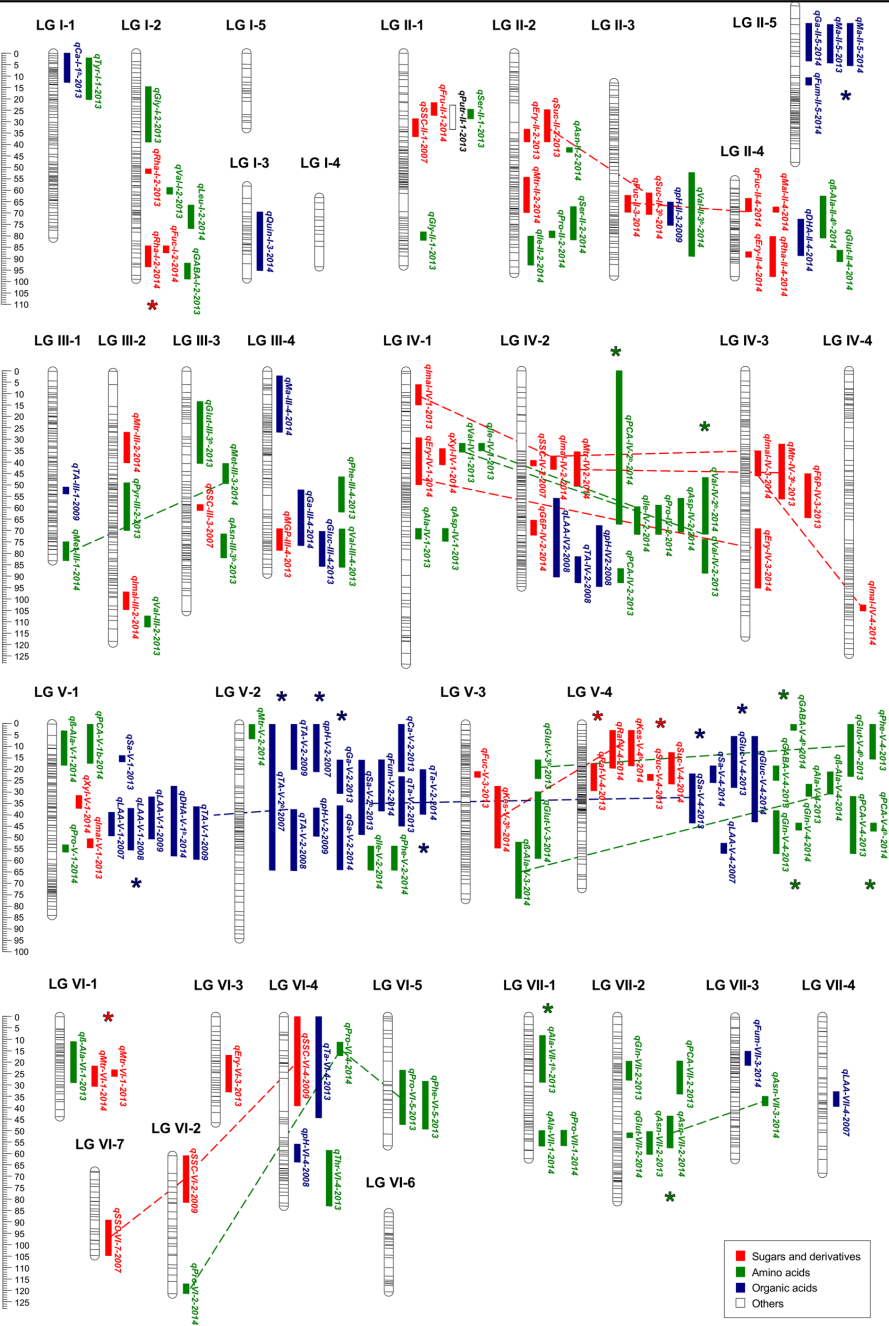
Positions of mQTL controlling primary metabolites and QTL for SSC, TA, and pH detected in the ‘232’ × ‘1392’ F_1_ population. 2-LOD QTL intervals are drawn at the right of each linkage group. Names of mQTL as described in Supplementary Table 3. Stable QTL, detected in different years, are highlighted with asterisks. Putative homoeo-QTL are joined by discontinued lines

mQTL analyses using the 50 identified primary metabolites were performed using the integrated map previously generated for the ‘232’ × ‘1392’ population^47^. This linkage map comprises 2089 SNP and SSR markers distributed into 33 linkage groups (LGs) corresponding to the full complement of 28 chromosomes. The map spans a total length of 2489 cM and the average distance between markers is 1.34 cM^47^. mQTL were analyzed using average metabolite values, from each year separately, for each of the 50 compounds. Other previously reported characters related to fruit quality, namely SSC, TA, pH, and L-AA content quantified in 2007, 2008, and 2009 in the same population, were also used for QTL analysis, since the current linkage map covers the strawberry genome with higher density of markers than previous maps used for these characters^26^. A total of 155 significant associations for a total of 47 traits were found between markers and phenotypes using restricted multiple QTL mapping (rMQM; Supplementary Table 3; Fig. 3). The majority of marker-trait associations were also detected by the Kruskal–Wallis test (*P* < 0.005). If more than one QTL for a given trait was detected in different years in approximately the same chromosomal regions, they were considered to be the same. Thus, the 155 QTL could be summarized into 133 unique QTL. From these, 113 QTL were detected 1 year and 20 (12.9%) QTL were stable over the two or three assessed years (Supplementary Table 3). QTL were identified across the seven homoeology groups (HGs) of the ‘232’ × ‘1392’ map, except in the three short LGs (Fig. 3). The number of QTL detected for each trait ranged from one (i.e., for glucose-6P, maltose, tyrosine, threonine, leucine, methyl-glucopyranoside, putrescine, pyruvic acid, quinic acid) to seven (for proline). The phenotypic variation (*R*^2^ in %) explained by each QTL ranged from 9.6% (for *qMtr-IV-2* in 2014) to 46.1% (for *qImal-IV-4* in 2014). Interestingly, clusters of QTL were detected in all HGs suggesting linkage or pleiotropic effects of loci. The largest clusters of QTL were found in HG V: One cluster on LG V-2 involved QTL for TA, pH, and citric, succinic, fumaric, glyceric, and threonic acids, with four of them stable over 2 years. Another cluster of QTL was detected on LG V-4 and comprised 10 QTL for three sugars (raffinose, kestose, and sucrose), two organic acids (succinic and glucuronic acids), and five amino acids (GABA, alanine, β-alanine, glutamine, and phenylalanine), with 50% of them being detected over 2 years. Surprisingly, several clusters of QTL involved primary metabolites that were significantly correlated (Supplementary Table 2; Fig. 2), i.e., QTL for organic acids and sugars in LG V-4 (glucuronic and succinic acids; raffinose and sucrose). Similarly, QTL for the coupled amino acids, alanine and glutamine, methionine and β-alanine, asparagine and glutamic acid, which were highly correlated (Fig. 2), co-located on LG V-4, LG VI-1, and LG VII-2, respectively. Other examples of QTL for correlated metabolites were detected for malic and glyceric acid in LG II-5, for sugars erythritol and sucrose on LG II-2, and for fucose and sucrose in LG II-3 (Supplementary Table 3; Fig. 3).

Putative homoeo-QTL, defined as QTL positioned in overlapping regions of different LGs belonging to the same HG, were identified for a number of sugars, acids and amino acids. The putative homoeo-QTL was detected the same year, such as one for fucose in 2014 on LG II-3 and II-4, or in different years such as for proline on LG VI-4 and VI-5. The highest number of homoeo-QTLs were detected in HG IV and V. QTL for isomaltose were detected in the four LGs of HG IV, with one QTL in 2013 and three in 2014. On HG V, three putative homoeo-QTL were detected during the first year for succinic acid in LG V-1, V-2, and V-4.

### Association of QTL, mQTL, and genic-markers controlling the variation in acids and sugars

Reanalysis of data obtained for SSC in 2007, 2008, and 2009^26^ in combination with the primary metabolites, identified a co-localization of mQTL for fructose and a QTL for SSC on LG II-1, with homoeo-QTL for related sugars such as sucrose, erythritol, and fucose on LG II-2, II-3, and II-4 in different years (Fig. 3). Different homoeo-QTL were detected for SSC on LG VI-2, VI-4, and VI-7, while an mQTL was detected in both years for maltotriose on LG VI-1 and in 2013 for erythritol on VI-3.

**Fig. 4:**
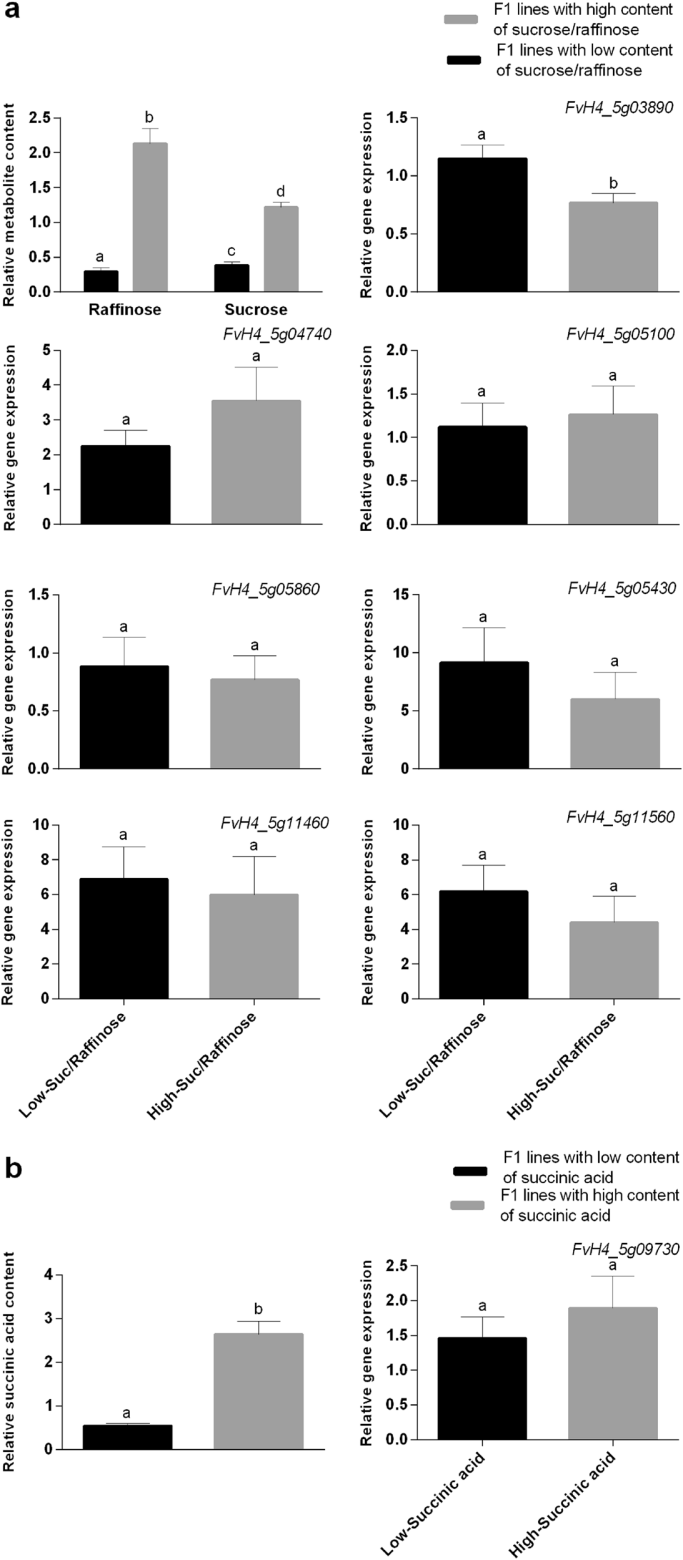
Expression analysis of candidate genes for sucrose, raffinose, and succinic acid content. Expression by quantitative real-time PCR (qRT-PCR) of candidate genes for the major mQTL detected in LG V-4 for sucrose and raffinose (**a**), and succinic acid (**b**). **a** The first graph depicts average metabolite content in two pools of selected F_1_ lines contrasting in raffinose and sucrose content in the 2 years. Subsequent graphs show expression levels by qRT-PCR in selected pools for *FvH4_5g03890: glucose-6-P 1-epimerase*, *FvH4_5g04740*: *UDP-glucose 4-epimerase*, *FvH4_5g05100*: *probable threhalose-phosphate synthase*, *FvH4_5g05430*: *triose phosphate translocator*, *FvH4_5g05860*: *galactinol-sucrose galactosyl-transferase-like*, *FvH4_5g11460*: *probable trehalase*, *FvH4_5g11560*: *bidirectional sugar transporter sweet4*. **b** Average content of succinic acid in two pools of selected F_1_ lines contrasting in this metabolite in the 2 years and expression level of candidate gene *FvH4_5g09730*, *succinate dehydrogenase assembly factor* in selected pools. The bars represent the mean expression value of five lines with low (l-Suc/Raffinose; l-succinic acid) and five lines with high (H-Suc/Raffinose; H-succinic acid) content of sucrose/raffinose and succinic acid, respectively. Error bars indicate ± SE. Different letters indicate significant differences between pool of lines using *t*-Student significant difference test adjusted to 95% significance

A major mQTL for sucrose (*qSuc-V-4*) and for raffinose (*qRaf-V-4*), controlling 22–30% of the variation, was detected in 2013 and 2014 in approximately the same position on LG V-4. The 2-LOD confidence interval for these mQTL expands a region of about 13 cM, from DArTseq marker 10028585 at position 13 cM to DArTseq marker 10016831 at 26 cM (Supplementary Table 4a). The orthologous region flanked by these markers in the *F. vesca* reference genome spans the interval 1,822,882–7,927,246 bp on chromosome 5 and includes a total of 1097 genes (Supplementary Table 4b). Among them, we found twelve candidate genes based on their annotated function related to sugar biosynthesis, metabolism, or transport (Supplementary Table 5). Seven of these genes, based on their putative function and their expression in red fruits (achenes and receptacle) of *F*. × *ananassa* cv. Camarosa^48^, were selected for qRT-PCR analysis, and are indicated in bold in Supplementary Table 5. To propose putative candidate genes in this region, we focused on those with functions related to sugar metabolism and showing expression in fruit according to the data previously published^48^. This reduced the gene list to seven genes (*FvH4_5g03890*, *glucose-6-P 1-epimerase; FvH4_5g04740, UDP-glucose 4- epimerase; FvH4_5g05100, threhalose-phosphate synthase; FvH4_5g05430, triose phosphate translocator; FvH4_5g11560, bidirectional sugar transporter sweet4; FvH4_5g11460, probable trehalase; FvH4_5g11560, bidirectional sugar transporter sweet4*). Using quantitative RT-PCR, we analyzed the basal expression of these candidate genes in ripe fruits from F_1_ lines showing contrasting sucrose and raffinose levels, including the parental lines and ten progeny lines. This analysis showed higher expression (~35%) of gene *FvH4_5g03890*, *glucose-6-P 1-epimerase* in those lines containing about fourfold lower sucrose and raffinose levels compared to lines with high levels of these sugars (Fig. 4a). In agreement, the percentage of variation explained by the QTL for raffinose and sucrose on LG V-4 ranged from 22.3 to 30.7% depending on the year.

Regarding TA, pH and acids, co-locations of QTL for fruit acidity traits with mQTL for glyceric, succinic, fumaric, citric, and threonic acids were detected on LG V-2 (Fig. 3). This result suggests the presence of a common QTL controlling the accumulation of these different acids and, as a consequence increasing TA and lowering the pH of fruits. Variation in these traits is provided by the male parent, with one allele increasing fruit acidity and the other reducing it (Supplementary Table 3). A homoeo-QTL for succinic acid was detected in 2013 and 2014 on LG V-4, but in this case the alleles affecting the variation in succinic acid are derived from the female parent ‘232’ (Supplementary Table 3). This stable mQTL, *qSa-V-4*, controls 19–28% of the phenotypic variation in succinic acid content depending of the year, and the 2-LOD confidence interval (18.18–25.6 cM) overlapped with that of *qSuc-V-4* and *qRaf-V-4* for sucrose and raffinose content (Fig. 3). Furthermore, the allelic effects increasing and decreasing the content of the three metabolites are inherited from the ‘232’ line, and in the same phase (i.e., QTL alleles modify all three metabolites in the same direction; Supplementary Table 4). This opens the possibility that a common gene with pleiotropic effects on sucrose, raffinose and succinic acid could be responsible for these changes. Alternatively, another gene related to succinic acid metabolism, *FvH4_5g09730*, a mitochondrial *succinate dehydrogenase assembly factor*, was found within the confidence interval of the QTL *qSa-V-4*. However, no significant differences in the expression of this candidate gene, encoding an enzyme catalyzing a TCA intermediate, was observed between lines with low and high content of succinic acid (Fig. 4b). Similar to the other candidate genes for sugars that were not differentially expressed, this result highlight the limitation associated with the fact that expression differences are not the only impactful genetic variation. Also, amino acid changes affecting enzymatic activity can contribute to the observed variation in metabolites.

### *Mannose-6-P-isomerase* is a candidate gene for variation in L-AA content

Ripe fruit of ‘232’ and ‘1392’ lines contain an average of 37.1 and 48.1 mg/100 g FW, respectively^26^. Using natural variation in L-AA content in the derived F_1_ progeny, three QTL explaining a total of 45% variation were previously identified^26^. Here, re-analysis of these stored data using the high density integrated map resulted in similar results (Fig. 3; Supplementary Table 3). The most important QTL for L-AA, *qLAA-V-1*, was detected in a narrower chromosomal interval than when using the previous maps and co-located with an mQTL for dehydroascorbic acid, *qDHA-V-1*, detected 1 year just below the threshold. The QTL *qLAA-IV-2*, previously detected in 2 years was now detected just 1 year and the third QTL, *qLAA-VII-4*, was detected the same year at the same position (although previously the LG was only 11.1 cM and was named LG VII-M1^26^). Candidate genes for the three QTL were previously identified based on orthologous positions in the *F. vesca* reference genome^26^. However, the confidence interval for the major QTL *qLAA-V-1* spanned a larger chromosomal region and varied among the three assessed years in the previous map. In this study, the phenotypic variation explained by *qLAA-V-1* varied from 27.7 to 35.5% depending on the year and the overlapping 2-LOD confidence interval was reduced to a region of 10 cM from DArTseq marker 10010698 at 38.6 cM to marker 10002629 at 48.8 cM (Supplementary Table 4a). The region flanked by these two markers in the *F. vesca* reference genome spans an interval of chromosome 5 from 8,160,286 to 13,129,181 bp and includes a total of 732 genes (Supplementary Table 4b). A search for genes involved in L-AA biosynthesis and recycling identified two candidate genes in the interval: (1) gene *FvH4_5g20650* with similarity to the *myo-inositol oxygenase* (*FaMIOX*) gene previously described^28^ and (2) gene *FvH4_5g21090* with similarity to a *mannose-6-phosphate isomerase* (*M6PI*) from *Arabidopsis*^49^.

To validate these genes and/or identify additional candidate genes underlying the three mQTL affecting L-AA content in strawberry, we identified differentially expressed genes (DEG) between two bulked RNA samples from F_1_ lines contrasting in L-AA content (Supplementary Figure 1). First, transcriptomes were obtained by Illumina sequencing using three biological replicates of each pool. An average of 33.97 million reads was generated for each sample, ranging from 30.83 to 36.75 M. An average of 32.79 M read-pairs passed the filter cutoff and 79.49% were mapped to the reference *F. vesca* v4.0.a1 genome assembly and annotation^43^. Analysis of differential expression detected 826 DEGs between contrasting lines, with 244 and 582 transcripts upregulated and downregulated in the high L-AA pool, respectively (DEG significance threshold fixed at *q*-value = 0.005; Supplementary Table 6). Eight and seven transcripts were not expressed in the low and high L-AA pool, respectively. The majority of these transcripts were shorter than 500 bp and with unknown function. For the rest of the DEGs, the ratios (log2 fold change) of differential expression ranged from −3.45 to 2.76, with negative and positive values indicating upregulation and downregulation in the high L-AA pool, respectively. The transcript with the highest difference in expression between the pools corresponds to the *F. vesca* gene *FvH4_5g21090*, with homology to the *M6PI*^49^. This locus lies within the confidence interval of *qLAA-V-1* and was therefore identified above as a candidate gene (*FaM6PI1* from now on). The expression of *FaM6PI1* in the high L-AA pool was 2.89 FPKM and ~11-fold higher (3.45 log2-fold change) than in the pool of fruits with low L-AA content (0.26 FPKM; Supplementary Table 6). The other candidate gene previously identified in the chromosomal interval, gene *FvH4_5g20650* with similarity to the *myo-inositol oxygenase* (*FaMIOX*), was not differentially expressed between the pools. Among the 582 transcripts with lower expression in the high L-AA pool, three strawberry transcripts with homology to *ascorbate oxidase* (*AO*) were identified, although none of them were within the confidence interval of any of the mQTL for L-AA. An increase in L-AA content has been reported in tomato after reducing *AO* expression using RNAi^50^. In addition, the expression of two transcripts with high similarity to *Arabidopsis* genes encoding enzymes catalyzing two consecutive reactions in the L-AA biosynthetic pathway in animals was lower in the high L-AA pool. These genes correspond to *FvH4_5g14230*, with high homology to the *UDP-glucose-dehydrogenase AT5G15490* and *FvH4_2g28000*, with the highest similarity to the *UDP-sugar pyrophosphorylase* (*AT5G52560*). Both enzymes belong to the KEGG ascorbic acid and aldarate metabolism pathway in *Arabidopsis*.

**Fig. 5:**
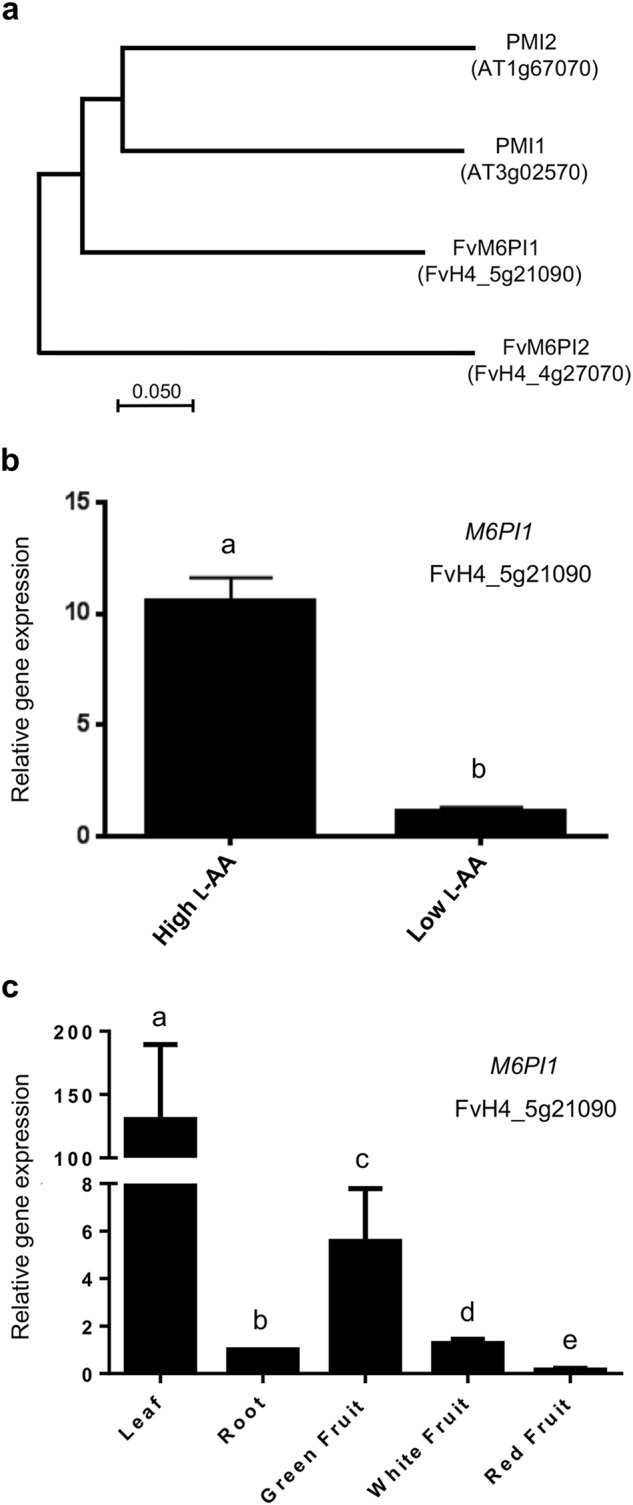
Molecular characterization of *FaM6PI1*, a candidate gene for *qLAA-V-1*. **a** Unrooted neighbor-joining phylogenetic tree of *Arabidopsis* AtPMI1 and AtPMI2 and *F. vesca* FvM6PI1 and 2 deduced proteins. The tree is drawn to scale, with branch lengths in the same units as those of the evolutionary distances used to infer the phylogenetic tree. The evolutionary distances were computed using the Poisson correction method in MEGA7 and are in the units of the number of amino acid substitutions per site. **b** Expression of *FaM6PI* in the high and low L-AA pools by qRT-PCR. Error bars indicate + SD. Different letters indicate significant differences between pool of lines using *t*-Student significant difference test adjusted to 99.9% significance level. **c** Expression level of *FaM6PI* in different tissues and during fruit ripening by qRT-PCR. Error bars indicate standard deviations from three biological replicates. Expression levels are expressed as a ratio relative to one of the samples. Different letters indicate significant differences between tissues using *t*-Student significant difference test adjusted to 95% significance level

There are two *M6PI* genes in *Arabidopsis*, *phosphomannose isomerase 1* and *2* (*AtPMI1* and *AtPMI2*) and the deduced proteins contained 432 and 441 amino acids, respectively, and showed 64% identity^49^. Similarly, a BLAST search in the *F. vesca* reference genome identified two genes with similarity to *Arabidopsis* *AtPMI* genes, the previously identified *FvM6PI1* (*FvH4_5g21090*) and *FvM6PI2 (FvH4_4g27070*). A phylogenetic analysis of *Arabidopsis* proteins and FvM6PI1 and FvM6PI2, which contained 434 and 437 amino acid residues, respectively, is shown in Fig. 5a. The similarity of the deduced protein encoded by the locus identified in the confidence interval of QTL *qLAA-V-I* (*FvH4_5g21090*; *FvM6PI1*) was higher than that of *FvM6PI2* to both PMI1 and PMI2. Furthermore, the consensus sequence YXDXNHKPE, typical of eukaryotic type I M6PI was present in both *F. vesca* protein sequences, and it was identical in PMI1 and FvM6PI1 (YKDDNHKPE), while PMI2 and FvM6PI2 possessed the consensus sequence YRDNNHKPE.

We next validated the differential expression of *FaM6PI1* observed by RNA-seq using qRT-PCR which resulted in the same ~11-fold higher expression in F_1_ lines with high L-AA (Fig. 5b). Quantitative RT-PCR also validated the RNA-seq data for five other genes (data not shown) resulting in a high correlation between RNA-seq and qPCR results (*R*^2^ = 0.928). Analysis of the expression of *FaM6PI* genes in the available transcriptome of the cultivar Camarosa^48^ indicated that both genes are expressed in all studied tissues (achenes and receptacle during ripening, leaves, and roots) with higher expression in green tissues. The expression of both genes was downregulated during ripening of achenes and receptacle and the expression of *FaM6PI1* was 10-fold lower than that of *FaM6PI2* in the majority of studied tissues (Supplementary Table 7). The expression profile of *FaM6PI1* in leaf, root, and during fruit ripening was very similar to the RNA-seq data when analyzed by qRT-PCR (Fig. 5c). Thus, expression of *FaM6PI1* was higher in green tissues.

## Discussion

In recent years, there is increasing interest in breeding better tasting strawberries while maintaining current yields. Key traits for improvement are sugar content and fruit acidity, which are measured by breeding programs as the aggregate parameters SSC and TA. These traits have variable heritability, environmental effects and GxE interactions in different fruit crops^25,51,52^, which complicates breeding initiatives to improve taste. A better understanding of the genetic control of individual metabolites will provide knowledge of which particular metabolite affect these traits, and what would be of help for the selection of markers for breeding new high quality strawberry cultivars. This study identified numerous mQTL for primary metabolites accumulating in ripe strawberry fruit. While several studies have used broad genetic crosses to identify mQTL of primary metabolites in other species such as tomato^12,53–56^, in strawberry significant research has been focused on defining QTL and genes controlling volatile organic compounds^27,36–38^. In contrast, the levels of a reduced number of primary metabolites were evaluated in different strawberry breeding populations^25,26,57,58^. Here, we have quantified the relative levels of 50 primary metabolites in a well characterized F_1_ population, providing the first insights into the genetic control of individual sugars, organic and amino acid content in strawberry fruits. The majority of mQTL for primary metabolites were detected in only one of the assessed years, indicating that primary metabolite content is affected to a large extent by the environment. This result contrasts with that observed in the same population and similar growing conditions, for volatile compounds, where about 50% of the mQTL were detected in at least two of three assessed years^27^. Other QTL studies for fruit quality traits in strawberry reported stability across different years of 27 and 36.4%^25,26^. However, previous studies considered a QTL stable when detected in at least 2 of 3 years, in contrast to the present study in which the majority of metabolites were only evaluated during 2 years.

Analysis of mQTL for primary metabolites from the perspective of their genomic location revealed that they were spread across the genome. However, there were a few hot spots for mQTL, particularly notable loci include those on LG IV-2, V-2, and V-4. Clustering of QTL have been often reported in several plant species, including strawberry, where loci controlling sugars and acids co-located on LGs belonging to HG VI^25,58^. In our study, a QTL for SSC and one for threonic acid co-located on LG VI-4. In addition, QTL for different sugars and acids co-located in LG V-4 in this work. In other studies, QTL for sugars and acids were also identified at approximately the same chromosomal interval on the same HG V, indicating that common loci may be controlling the variation in multiple genetic backgrounds^25,57,58^. In our study, QTL for sugar traits were detected in the middle of LGs belonging to HG V as previously described^25,58^. Similarly, other QTL for sugars were detected in the upper half of LGs of HG VI in our study and also in three other reports^25,57,58^. For acidity, putative common QTL across the four studies were detected on the lower part of LGs belonging to the HG IV. Homoeo-QTL for acidity traits detected in our study in three LGs of HG V could be common with QTL detected in other studies for TA^57^ and TA, pH, citric acid, and malic acid^25^. A major QTL for glyceric and malic acids detected on the upper half of LG II-5 could overlap with regions where QTL for TA and citric acid were mapped in other studies^25,57^. These QTL common to different backgrounds provide a higher chance of identifying reliable markers for marker-assisted breeding in key traits for fruit quality.

Sucrose levels increase dramatically during strawberry fruit ripening^1^. This rise may be due to photosynthate translocation from the leaf, where it is loaded into the phloem in either an apoplastic or a symplastic manner^59,60^. Here, mQTL for the sugars sucrose and raffinose and for succinic acid co-located at the same chromosomal region on LG V-4. We have to consider the possibility that raffinose is one of the main oligosaccharides constituting a significant component of phloem-transported sugars, as reported in certain species such as cucurbit plants^61^. In the fruit flesh of watermelon, raffinose levels are negligible, due to its hydrolysis while being unloaded^61^. This could be also the case in strawberry fruit. In other reports, the accumulation of raffinose has been associated with stressful environmental conditions^62,63^. In our study, the three metabolites, sucrose, raffinose, and succinic acid, were correlated in both years and the variation for them was associated with positive and negative alleles provided by the ‘232’ line, which presented lower amount of these metabolites in fruits (Table 1) and also lower values of total sugars (estimated as SSC) and TA^26^. However, whether the variation of these metabolites is controlled by a gene with pleiotropic effects, or two linked genes, will need further investigations.

Two QTL on LG V-4, controlling sucrose and raffinose content, shared a common interval that contained a gene encoding *glucose 6-P 1 epimerase* (ortholog of Solyc01g095470 in tomato). As aldose 1-epimerase is catalyzing the interconversion of alpha-anomers and beta-anomers of sugars such as glucose and galactose^64^, this gene appears to be a strong candidate gene for this QTL based in position and differential expression in contrasting lines. However, it remains to be functionally validated. Interestingly, in addition to the enzymatic activity, this protein has been suggested to participate in stress signal regulation by playing an important role in the early development of tolerance under stressful conditions^65^. As exciting as these results might seem, limitations in the selection of candidates include (i) the assumption that the causal mutation must affect transcript expression in a gene already annotated for sugar metabolism, (ii) the relatively small sample size for analysis of differential expression and (iii) the moderate contribution of this QTL to the total variance of the trait in selected contrasting lines with extreme phenotypes, which most probably gather positive and negative alleles for different QTL contributing to the variation in sucrose and raffinose.

Succinate dehydrogenase (*gene FvH4_5g09730)* is a key protein in the TCA cycle, and catalyzes the oxidation of succinic acid to fumaric acid^9,66^. This gene, localized in the confidence interval of mQTL *qSa-V-4*, appear to be a strong candidate for the QTL controlling succinic acid content. This must prompt future studies to determine whether *FvH4_5g09730* is the gene underlying this QTL.

In this study, the majority of sugars were positively correlated with the majority of acids, and thus fruits with higher content of acids also had higher content of sugars. However, two previous studies^26,67^ reported no correlation between SSC and TA and one of these studies found a low but significant correlation between pH and SSC^26^. According to Lerceteau-Köhler et al.^25^, positive and negative correlations were observed between pH and SSC, and between TA and SSC, respectively. Taken together, these results suggest that higher sugar content could be improved in parallel with fruit acidity for loci in the same chromosomal regions when positive alleles are in coupling phase or independently when in different chromosomal regions, particularly by targeting different sugar and acid metabolites.

A total of four well characterized and proposed alternative pathways for L-AA biosynthesis have been described in plants: d-mannose/l-galactose, galacturonate, *myo*-inositol, and l-gulose pathways^68,69^ The prevalent pathway in plants uses d-glucose-6-P as the initial precursor and GDP-d-mannose and GDP-l-galactose as intermediates (mannose/galactose pathway)^70^. In *Arabidopsis*, two M6PI isoenzymes, AtPMI1 and AtPMI2 have been described, which catalyze the reversible isomerization between d-fructose-6-P and d-mannose-6-P^49^.This enzymatic step precedes the synthesis of the intermediate GDP-d-mannose, thus connecting d-fructose-6-P to L-AA biosynthesis. Both genes are constitutively expressed, however *PMI2* is expressed at a lower level, and RNAi and analysis of mutant lines indicated that PMI1, but not PMI2, is involved in L-AA biosynthesis in *Arabidopsis*^49^. In this study, we have identified *FaM6PI1* from strawberry as the most similar gene to the *Arabidopsis* AtPMI1 and as a candidate gene for the locus controlling L-AA content on LG V-1. The expression of the gene was about 11-fold higher in ripe fruits of lines with higher L-AA content. L-AA content in strawberry fruits increases during development and ripening, from <20 mg/100 g FW to reaching an average concentration of about 50 mg/100 g FW in ripe fruit^28^. Analysis of *FaM6PI1* expression during ripening has shown that the gene is predominantly expressed in leaves, at a lower level in green fruits, and it is downregulated during fruit ripening. Other genes encoding enzymes of the mannose/galactose pathway are also downregulated as the fruit ripens^28^. These expression results support this pathway as responsible for L-AA biosynthesis in green fruits. If *FaM6PI1* is the underlying gene for *qLAA-V-I*, our data indicate that biosynthesis of L-AA, from d-fructose-6-P, at early stages of ripening is also important for the final L-AA concentration at the ripe stage.

In parallel, lines with higher L-AA content were characterized by reduced expression of enzymes transforming d-glucose-1-P to UDP-d-glucose and to UDP-d-glucuronate, enzymatic steps common to the animal L-AA pathway^68^ and that drive d-glucose-6-P away from the mannose/galactose biosynthetic pathway. Therefore, a reduced expression of these enzymes may increase the pool of d-glucose-6-P available for biosynthesis of L-AA through the prevalent pathway in plants. In addition, the expression of three genes with homology to *AO*s was significantly lower in F_1_ lines with higher L-AA content, contributing to maintaining the L-AA pool in a reduced state (Supplementary Table 6).

In conclusion, the data presented here complement and extend that previously documented for fruit quality and volatile traits^26,27^. Our data highlighted metabolic networks of primary metabolites in strawberry fruits, suggesting related biological pathways. In general, correlated metabolites were controlled by mQTL in overlapping chromosomal intervals. Furthermore, comparison to other studies identified common genomic regions and, presumably, common genes in different populations, which may control the variation of these traits. In term of directed metabolic engineering strategies, several major mQTL were identified that could be used for designing breeding strategies to improve the nutritional quality of strawberry. In particular, we focused on QTL for raffinose, sucrose, succinic, and ascorbic acids that explained a large proportion of the phenotypic variation and were also stable in all assessed years, being potential candidates for future marker-assisted selection. In this regard, a number of candidate genes have been identified and their expression characterized; however, future functional studies using transgenic approaches will validate the involvement of these candidate genes and help in enhancing fruit metabolite content. Evaluation of global gene expression levels on these F_1_ lines would likely both deepen our understanding of the molecular basis of the QTL described in this study, as well as would hasten the improvement of fruit nutritional quality in strawberry.

## Materials and methods

### Plant material

The octoploid strawberry mapping population used in this study consists of a full-sib family of 95 F_1_ individuals derived from an intraspecific cross between the breeding lines ‘232’ and ‘1392’^26^. The two parental lines were chosen among the breeding lines from Instituto Andaluz de Investigación y Formación Agraria y Pesquera (IFAPA) because they differed in important agronomical and fruit quality traits; ‘232’ is a very productive strawberry (*Fragaria* × *ananassa*) line, whereas ‘1392’ has firmer and tastier fruits. Six plants of each F_1_ and parental lines were vegetatively propagated and grown under commercial conditions in Huelva (Spain) during two consecutive years (2013–2014). For ascorbic acid (L-AA), SSC, TA, and pH analysis, the data were obtained from years 2007, 2008, and 2009^26^. The mapping population was grown under macro tunnels of polyethylene following conventional practices with an inter-row distance of 30 cm and a distance between plants of 25 cm.

### Metabolite profile analysis

Metabolite profiles were obtained by GC-time of flight-(TOF)-MS (as described below) from fruits harvested the same day in the middle of the spring/winter season in the consecutive years 2013 and 2014. For each line, 20–25 fully ripe fruits were harvested, pooled into three biological replicates immediately frozen in liquid nitrogen, and stored at −80 °C until analysis. After grinding fruit samples in liquid nitrogen, the relative levels of metabolites were determined from frozen samples following the protocol previously established^42^.

### Statistical analysis

Normality of trait distributions was evaluated by the Shapiro–Wilk test. For most metabolites deviating from normality (*P* < 0.05), a number of transformations (Ln, square root, inverse of square root, square, cube, reciprocal, and arcsine) were tested and the transformation that gave the least-skewed result was used to perform QTL analysis. Correlation analysis based on Pearson correlation was performed using R software.

### Linkage mapping and QTL analysis

For mQTL detection we used the integrated map previously developed for the ‘232’ × ‘1392’ mapping population^47^. The ‘232’ × ‘1392’ map contains a total of 2089 SNP and SSR markers spanning a total length of 2489 cM. QTL analyses were performed using MapQTL 5^71^. The population was derived from two heterozygous parents with linkage phases originally unknown and was coded under the population type ‘cross pollinated’ (CP), thus four genotypic classes were modeled (ac, ad, bc, and bd). The raw relative data were analyzed first by the nonparametric Kruskal–Wallis rank-sum test. A stringent significance level of *P* ≤ 0.005 was used as a threshold to identify markers linked to QTL. Second, the integrated genetic linkage map and transformed data sets for non-normally distributed traits were used to identify and locate mQTL using interval mapping (IM) with a step size of 1 cM and a maximum of five neighboring markers. Significance LOD thresholds were estimated with a 1000-permutation test for each trait. The most significant markers were then used as co-factors for restricted multiple QTL mapping (rMQM) analysis. mQTL with LOD scores greater than the genome-wide threshold at *P* ≤ 0.05 were declared significant. Significant mQTL location and 2-LOD confidence intervals were drawn using MapChart 2.2.

### In silico candidate gene search

Physical map positions of DArT-derived SNPs and microsatellites used in this study were obtained by aligning the DArT sequences (Supplementary Table 4) and SSR primer sequences in the ‘232’ × ‘1392’ map^47^ to the most updated *F. vesca* v4.0.a1 genome assembly^43^ using Bowtie 2.1.0 as previously reported^47^. Chromosomal regions spanning the orthologous positions of markers in the 2-LOD confidence interval were investigated for candidate genes based on annotated biochemical functions.

### RNA extraction and qRT-PCR

Total RNA was extracted from strawberry fruits by differential precipitation with 2-butoxyethanol, as previously reported^72^. Before reverse transcription, RNA was treated with DNase I (Fermentas) to eliminate contaminating genomic DNA. First-strand cDNA synthesis was performed using 750 ng of RNA in a final volume of 20 μL using the iScript cDNA synthesis kit (Bio-Rad), according to the supplier’s protocol. Relative quantification of transcripts was analyzed by qRT-PCR using the SsoAdvance Universal SYBR Green Supermix (Bio-Rad). Relative quantification of the target expression level was performed using the comparative cycle threshold method. Expression data were normalized to the reference gene *FaGAPDH2*^73^ and *FaCHP1*^74^. Primers used are shown in Supplementary Table 8.

### RNA-seq from pooled samples and analysis of differential expression

For identification of genes differentially expressed between F_1_ lines contrasting in L-AA content, RNA was extracted as described above from two pools of ripe fruit from contrasting F_1_ lines. The high L-AA and low L-AA bulked pools consisted of an equivalent amount of fruit sample from 8 F_1_ lines each with high and low L-AA content, respectively. Ripe fruit were harvested and divided into three biological replicates, ground using liquid nitrogen, and stored at −80 °C until further analysis. RNA quantity and quality were determined based on absorbance ratios at 260 nm/280 and 260 nm/230 nm using a NanoDrop spectrophotometer (ND-1000 V3.5, NanoDrop Technologies, Inc.). The integrity of the RNA samples was assessed by agarose gel electrophoresis and further verified using a 2100 Bioanalyzer (Agilent, Folsom, CA), and RIN values ranged between 7.3 and 8.1 for the six samples.

Paired-end libraries with ~300 bp insert size were prepared for each sample and sequenced in a HiSeq2000 lanes using 2 × 100 bp reads. An average of 33.97 million reads was generated for each sample, ranging from 30.83 to 36.75 M. Raw RNA-seq reads were processed to remove low-quality nucleotides and aligned to the latest *Fragaria vesca* v4.0.a1 genome assembly^43^ using the program HISAT2 2.1.0^75^. Default parameters of HISAT2 were used, allowing 40 multiple alignments per read and a maximum of two mismatches when mapping reads to the reference.

The aligned read files were processed by Cufflinks v2.2 essentially as previously described^38^. Reads were assembled into transcripts, which were classified as known, corresponding to annotated genes (*Fragaria vesca* v4.0.a1 genome annotation^43^), or novel, their abundance was estimated, and tests for differential expression between the samples were performed. Normalized RNA-seq fragment counts were used to measure the relative abundances of transcripts measured as fragments per kilobase of exon per million fragments mapped (FPKM). Bioinformatics processes were developed at Supercomputing and Bioinnovation Center (SCBI) at Málaga (Spain).

## Electronic supplementary material

Supplementary Figure 1

Suppplementary Table 1

Suppplementary Table 2

Suppplementary Table 3

Suppplementary Table 4

Suppplementary Table 5

Suppplementary Table 6

Suppplementary Table 7

Suppplementary Table 8

## Data Availability

Illumina RNA-seq reads from high and low L-AA pools have been deposited at the European Nucleotide Archive (https://www.ebi.ac.uk/ena) under the study reference PRJEB25718.
