# Supplementary figures and images for: Identification of quantitative trait loci and candidate genes for primary metabolite content in strawberry fruit
Source: Hortic Res. 2019 Jan 1;6:4. doi: 10.1038/s41438-018-0077-3 (PMC6312544; doi:10.1038/s41438-018-0077-3)

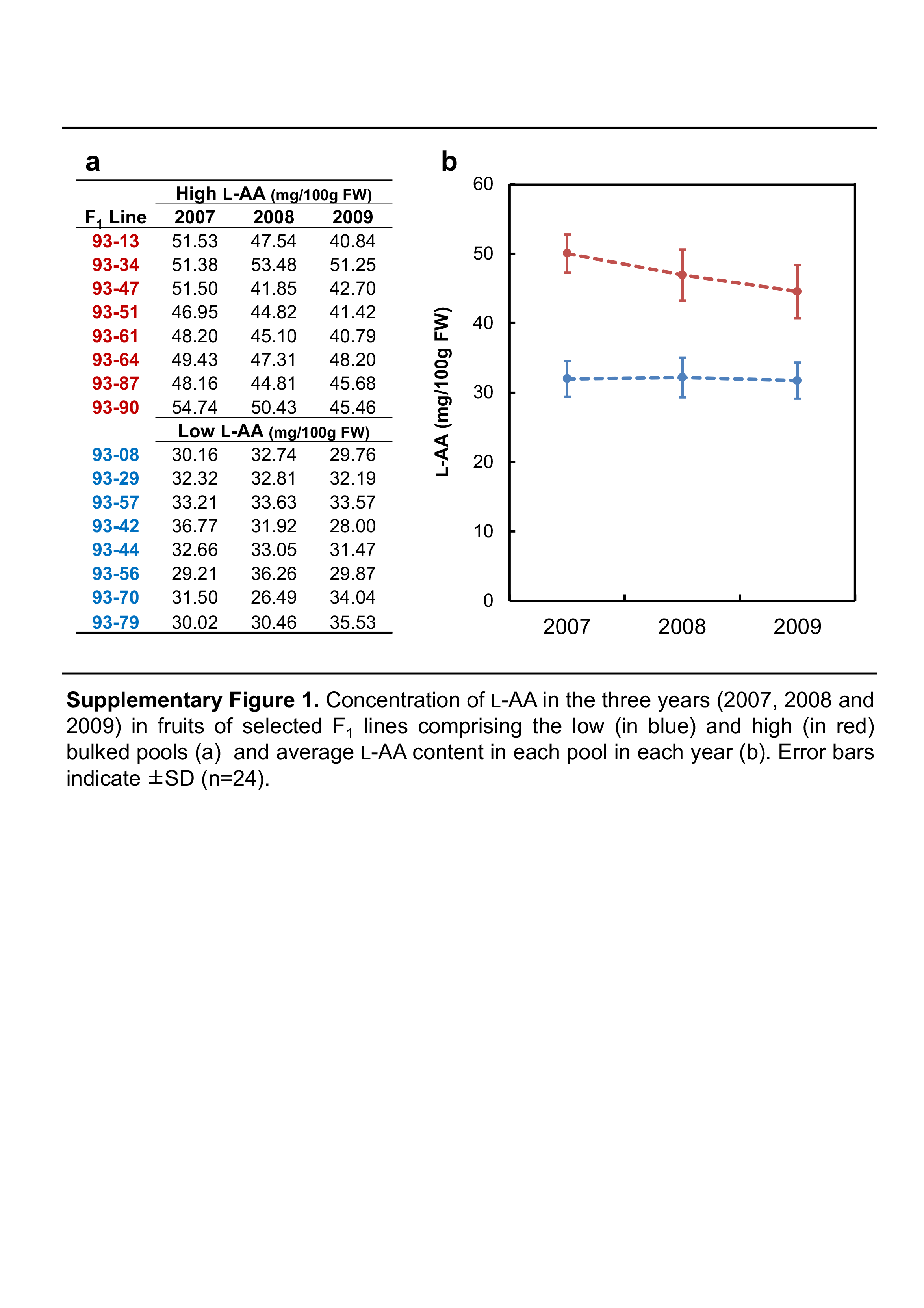

Supplement: Supplementary file 1 — Supplementary Figure 1 [file 41438_2018_77_MOESM1_ESM.tif]
